# Inverse planning for combination of intracavitary and interstitial brachytherapy for locally advanced cervical cancer

**DOI:** 10.1093/jrr/rrt072

**Published:** 2013-05-31

**Authors:** Kotaro Yoshio, Naoya Murakami, Madoka Morota, Ken Harada, Mayuka Kitaguchi, Kentaro Yamagishi, Shuhei Sekii, Kana Takahashi, Koji Inaba, Hiroshi Mayahara, Yoshinori Ito, Minako Sumi, Susumu Kanazawa, Jun Itami

**Affiliations:** 1 Department of Radiation Oncology, National Cancer Center Hospital, Tokyo, Japan; 2 Department of Radiology, Okayama University Hospital, Okayama, Japan

**Keywords:** cervical cancer, IPSA, combination brachytherapy, optimize, HDR

## Abstract

The main purpose of this study was to compare three different treatment plans for locally advanced cervical cancer: (i) the inverse-planning simulated annealing (IPSA) plan for combination brachytherapy (BT) of interstitial and intracavitary brachytherapy, (ii) manual optimization based on the Manchester system for combination-BT, and (iii) the conventional Manchester system using only tandem and ovoids. This was a retrospective study of 25 consecutive implants. The high-risk clinical target volume (HR-CTV) and organs at risk were defined according to the GEC-ESTRO Working Group definitions. A dose of 6 Gy was prescribed. The uniform cost function for dose constraints was applied to all IPSA-generated plans. The coverage of the HR-CTV by IPSA for combination-BT was equivalent to that of manual optimization, and was better than that of the Manchester system using only tandem and ovoids. The mean V_100_ achieved by IPSA for combination-BT, manual optimization and Manchester was 96 ± 3.7%, 95 ± 5.5% and 80 ± 13.4%, respectively. The mean D_100_ was 483 ± 80, 487 ± 97 and 335 ± 119 cGy, respectively. The mean D_90_ was 677 ± 61, 681 ± 88 and 513 ± 150 cGy, respectively. IPSA resulted in significant reductions of the doses to the rectum (IPSA D_2cm^3^_: 408 ± 71 cGy vs manual optimization D_2cm^3^_: 485 ± 105 cGy; *P* = 0.03) and the bladder (IPSA D_2cm^3^_: 452 ± 60 cGy vs manual optimization D_2cm^3^_: 583 ± 113 cGy; *P* < 0.0001). In conclusion, combination-BT achieved better tumor coverage, and plans using IPSA provided significant sparing of normal tissues without compromising CTV coverage.

## INTRODUCTION

Intracavitary brachytherapy (ICBT) plays a major role in the treatment of patients with cervical carcinoma [1–5]. When a tumor cannot be optimally encompassed by standard ICBT, interstitial brachytherapy (ISBT) is recommended to achieve better dose distribution [6].

In our hospital, combination brachytherapy (BT) of ICBT and ISBT has been performed to achieve even better dose distribution for advanced bulky lesions. However, an appropriate optimizing method for combination-BT has not been established. Until recently we performed manual optimization based on the Manchester system.

Inverse-planning simulated annealing (IPSA) is an optimization tool of high-dose rate brachytherapy (HDR-BT) that has been developed at the University of California, San Francisco [7, 8]. The optimal solution is obtained by minimizing the objective function through an iterative process. The algorithm uses fast simulated annealing to process the cost functions to arrive at an optimal solution in less than a minute. IPSA has been found to be superior with respect to target coverage and normal-tissue sparing compared with traditional optimization methods for prostate [9–13] and gynecologic malignancies [7, 14–16].

The main purpose of this study is to compare three different treatment plans for locally advanced cervical cancer: (i) the IPSA plan for combination-BT, (ii) manual optimization based on the Manchester system for combination-BT, and (iii) the conventional Manchester system using only tandem and ovoids.

## MATERIALS AND METHODS

**Fig. 1.**
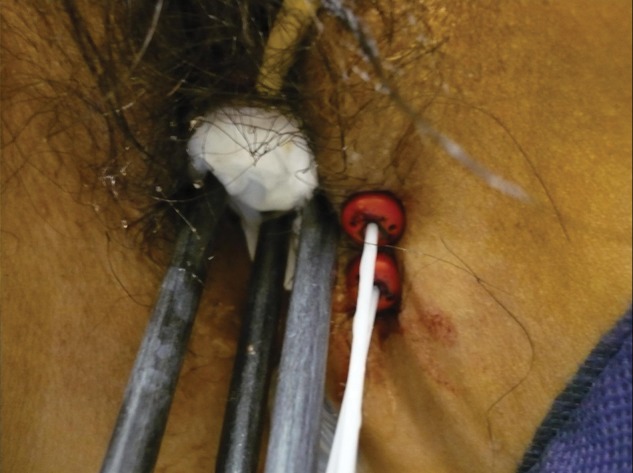
Two plastic interstitial needles are inserted from the left side of vulva. A CT-compatible tandem and ovoids are also inserted.

**Fig. 2.**
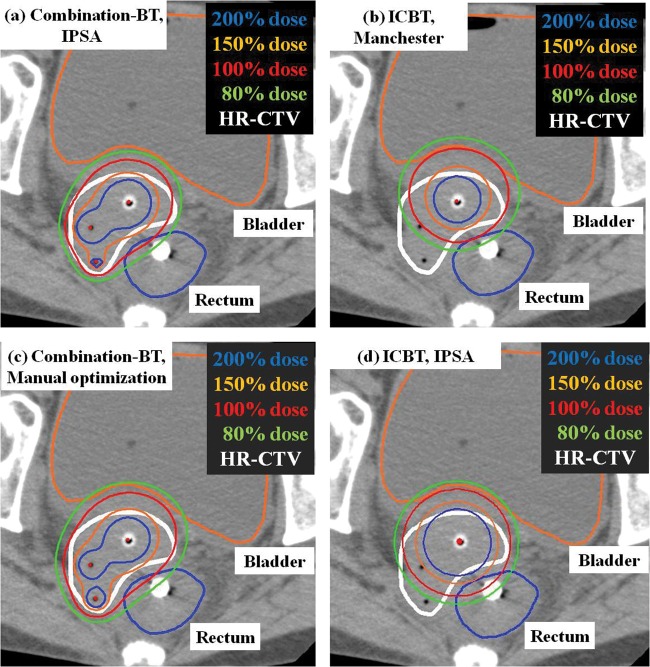
Comparison of dose distributions between three planning methods: (**a**) Inverse planning simulated annealing (IPSA) for combination-BT, (**b**) conventional Manchester system using tandem and ovoids, and (**c**) manual optimization based on the Manchester system for combination-BT, as well as (**d**) IPSA using tandem and ovoids.

This is a retrospective study of 25 consecutive implants (for nine patients) for primary cervical cancers. All patients selected for this study underwent conventional external radiation therapy and HDR-BT using combination-BT between August 2010 and March 2012. All patients were classified into Stage IIIB according to the International Federation of Gynecology and Obstetrics (FIGO) system. With external radiation therapy using 15-MV X-rays in a conventional fractionation, the whole pelvis was irradiated by up to 30 Gy, and the ensuing 20 Gy to the pelvic sidewall was administered with central shielding. Combination-BT was initiated upon the introduction of the central shielding. External radiation therapy was not performed on the day of BT. For combination-BT, CT-compatible tandem and ovoids were used with plastic interstitial needles (5F Proguide needles; Nucletron BV, Veenendaal, Netherlands). They were inserted under transrectal ultrasound guidance and fluoroscopy without a template (Fig. 1). Combination-BT was performed under caudal and local anesthesia. The interstitial catheters were placed to encircle the parametrial invasion. The distance between catheters was kept less than 2 cm, if possible. After the insertion, CT scans of 2-mm slice thickness were taken on an Aquilion LB CT scanner (TOSHIBA Medical Systems, Japan). After CT scanning, needle positions were changed if we could not make a favorable dose distribution, or if the needle position was close to the organs at risk (OARs). The depth of the needle was determined by CT images. Because the off-set length of plastic needle is 4 mm, we need to take the off-set length into consideration to make a favorable dose distribution. Treatment planning was based on post-implant CT imaging and was performed using Oncentra v4.1 (Nucletron, The Netherlands). A radiation oncologist delineated the high-risk clinical target volume (HR-CTV) using pretreatment clinical extent, imaging (pretreatment and during external radiation therapy, but just before the start of BT), intraoperative findings, and radio-opaque silver markers placed during the procedure. HR-CTV volumes were defined according to the GEC-ESTRO Working Group recommendation [17]. OAR volumes included the bladder, rectum, bowel and vagina. The rectal wall and its cavity were contoured from the anus to the rectosigmoid flexure. All bowel walls and their cavity surrounding the uterus and 2 cm above the fundus of the uterus were contoured. The vagina wall was drawn with 2-mm thickness. All treatments were performed with the plan produced by manual optimization based on the Manchester system with a prescribed dose of 6 Gy/fraction. All applicators were removed after combination-BT and the procedure was repeated for each session of combination-BT. All patients underwent four fractions of HDR-BT with a total prescribed dose of 24 Gy. For comparison with manual optimization, HDR dose distributions were created according to the different dose optimization methods as follows: the conventional Manchester system using only tandem and ovoids; IPSA using combination-BT; and IPSA using only tandem and ovoids. Figure 2 shows the dose distributions achieved by each planning method.

### Conventional Manchester system

For the production of manual optimization, conventional ICBT with only tandem and ovoids was made according to the Manchester system. In the Manchester system, the dose to point A was set to 6 Gy, and the relative contribution of tandem and ovoids to the point A dose was set to 1.8:1.

### Manual optimization based on the Manchester system

After the production of the conventional Manchester dose planning, the locations of dwell position were determined for the interstitial applicator based on CT images. Coronal and sagittal images were very useful. Each dwell position was set with 5-mm intervals. About 20% of the ovoid's dwell time was allocated to the interstitial applicators. Finally, fine-tuning was performed by manual graphical optimization to realize adequate dose coverage to the HR-CTV. Basically, dwell times of dwell points in each interstitial applicator were set up equally in this method. Point A was irradiated at more than 6 Gy in this method.

### IPSA

In IPSA, an optimal plan is sought that meets the dose objective parameters of both CTV and the OARs with the parameters' relative importance represented by weights. Both optimal dwell positions and dwell times are calculated. In this study, about 20-second sets of IPSA were repeated up to three times. Table 1 provides a representative set of dose objective parameters used to obtain an IPSA plan in this study. IPSA with combination-BT and ICBT used the same dose objective parameter. The weight is a factor reflecting the relative importance.

**Table 1.** Representative set of dose objective parameters used in IPSA

	Minimum (cGy)	Weight	Maximum (cGy)	Weight
HR-CTV surface	600	200		
HR-CTV volume	600	100		
Bladder surface			450	100
Rectum surface			450	100
Bowel surface			400	100

HR-CTV = high-risk clinical target volume, IPSA = inverse-planning simulated annealing.

### Plan evaluation

Dosimetric outcomes from the four different plans were compared. We analyzed the dose–volume histograms with the following endpoints: D_100_ and D_90_ for the minimum doses to 100% and 90% volumes of HR-CTV, and V_200_, V_150_ and V_100_ for the volumes of HR-CTV enclosed by 200, 150 and 100% of the prescribed dose of combination-BT. The volumes covered by 100% (V_PD_) and 200% (V_2PD_) of the prescription dose were also evaluated. Bladder, rectum, bowel and vagina were considered as OARs. For each organ, D_0.1__cm^3^_, D_1__cm^3^_, and D_2__cm^3^_ were calculated for the maximal doses to 0.1 cm^3^, 1 cm^3^ and 2 cm^3^ of the OARs, respectively. The homogeneity index (HI) was defined as (V100–V150)/V100. The differences between the parameters were compared for the four plans using analysis of variance (ANOVA), with resultant two-sided *P* values < 0.05 considered to indicate statistically significant differences.

## RESULTS

All 25 implants of the nine patients were evaluated. Their median age was 51 years (range, 43–63). The mean number of implanted interstitial needles was 2.8 (range, 2–6). The mean HR-CTV was 29.6 ml (range, 5.4–66.3).

### Dose and volume parameters for HR-CTV and V_PD_ (Table 2)

A summary of the dose and volume parameters for OARs (bladder, rectum, bowel and vagina) is shown in Table 2. Coverage of the HR-CTV with IPSA and manual optimization using combination-BT was significantly better than that with the conventional Manchester system of ICBT alone. HR-CTV coverage was the same for both IPSA and manual optimization of combination-BT with a mean V_100_ of 96 ± 3.7 and 95 ± 5.5%, respectively, a mean D_100_ of 483 ± 80 and 487 ± 97 cGy, respectively, and a mean D_90_ of 677 ± 61 and 681 ± 88 cGy, respectively. V_100_, D_100_ and D_90_ showed no significant difference between IPSA and manual optimization. In addition, despite the equivalent or better CTV coverage, the V_PD_ and V_2PD_ volumes obtained by the IPSA of combination-BT (mean V_PD_, 89 ± 33 and V_2PD_, 21 ± 8.6 ml) were significantly lower than those obtained by manual optimization (mean V_PD_, 131 ± 22 and V_2PD_, 30 ± 6.5 ml) or the Manchester system of ICBT alone (mean V_PD_, 116 ± 10 and V_2PD_, 29 ± 4.6 ml). V_150_, V_200_ and HI showed no significant difference between IPSA with combination-BT, manual optimization of combination-BT, and the Manchester system of ICBT alone. For reference, the dose–volume parameter achieved by IPSA using tandem and ovoids alone is also listed in Table 2.

**Table 2.** Dose–volume parameters of the conventional Manchester system, manual optimization, and IPSA

Parameters	(a) ICBT, IPSA (mean ± SD)	(b) ICBT, Manchester (mean ± SD)	(c) combination-BT, manual optimization (mean ± SD)	(d) combination-BT, IPSA (mean ± SD)	*P*-value (a) vs (b)	*P*-value (b) vs (c)	*P*-value (d) vs (b)	*P*-value (c) vs (d)
High-Risk CTV								
V_200%_	41.5 (± 10.8)	32.6 (± 12.2)	34.4 (± 12.8)	33.1 (± 10.3)	0.04	0.95	0.99	0.98
V_150%_	61.6 (± 11.1)	53.5 (± 15)	60.8 (± 15.1)	58 (± 12.6)	0.16	0.23	0.65	0.88
V_100%_	87 (± 7.9)	80 (± 13.4)	95 (± 5.5)	96 (± 3.7)	0.04	<0.0001	<0.0001	0.91
D_100_ (Gy)	356 (± 101)	335 (± 119)	487 (± 97)	483 (± 80)	0.88	<0.0001	<0.0001	0.99
D_90_ (Gy)	573 (± 117)	513 (± 150)	681 (± 88)	677 (± 61)	0.22	<0.0001	<0.0001	0.99
Rectum								
D_0.1 cm_3	638 (± 203)	662 (± 192)	624 (± 148)	519 (± 86)	0.95	0.63	0.003	0.03
D_1 cm_3	514 (± 153)	554 (± 151)	527 (± 116)	442 (± 73)	0.68	0.69	0.003	0.03
D_2 cm_3	463 (± 135)	503 (± 131)	485 (± 105)	408 (± 71)	0.6	0.82	0.006	0.03
Bladder								
D_0.1 cm_3	737 (± 161)	751 (± 161)	788 (± 222)	599 (± 104)	0.99	0.71	0.006	0.0005
D_1 cm_3	602 (± 122)	640 (± 132)	630 (± 126)	491 (± 66)	0.62	0.94	<0.0001	0.0001
D_2 cm_3	540 (± 106)	596 (± 122)	583 (± 113)	452 (± 60)	0.24	0.89	<0.0001	<0.0001
Bowel								
D_0.1 cm_3	524 (± 159)	876 (± 1279)	913 (± 1321)	480 (± 185)	0.54	0.01	0.1	0.08
D_1 cm_3	413 (± 114)	538 (± 249)	566 (± 264)	380 (± 113)	0.12	0.004	0.0003	0.001
D_2 cm_3	373 (± 99)	479 (± 180)	518 (± 183)	345 (± 105)	0.06	0.03	<0.0001	<0.0001
Vagina								
D_0.1 cm_3	1 566 (± 596)	1 267 (± 163)	1 294 (± 372)	1 096 (± 331)	0.04	0.7	0.03	0.02
D_1 cm_3	948 (± 320)	1 004 (± 150)	911 (± 141)	688 (± 151)	0.77	0.003	<0.0001	<0.0001
D_2 cm_3	623 (± 198)	865 (± 150)	763 (± 105)	524 (± 119)	<0.0001	0.0001	<0.0001	<0.0001
Volume parameter								
V_PD_	92 (± 56)	116 (± 10)	131 (± 22)	89 (± 33)	0.07	0.000 2	0.0002	<0.0001
V_2PD_	27 (± 19)	29 (± 4.6)	30 (± 6.5)	21 (± 8.6)	0.84	0.88	<0.0001	<0.0001
HI	0.29 (± 0.08)	0.34 (± 0.09)	0.36 (± 0.14)	0.39 (± 0.12)	0.38	0.93	0.3	0.67

IPSA = inverse planning annealing, CTV = clinical target volume, ICBT = intracavitary brachytherapy, SD = standard deviation, V_PD_ = the absolute volume covered by 100% of the prescription dose, V_2PD_ = the absolute volume covered by 200% of the prescription dose, HI (Homogeneity Index) is defined as (V100 – V150)/V100.

### Dose and volume parameters for OARs (Table 2)

The data suggest that IPSA with combination-BT leads to favorable sparing of the adjacent organs. The dose to the OARs obtained from IPSA with combination-BT (rectum D_2__cm^3^_: 408 ± 71 cGy, bladder D_2__cm^3^_: 452 ± 60 cGy, bowel D_2__cm^3^_: 345 ± 105 cGy) was generally lower than for that of the other plans.

## DISCUSSION

The results of the present study have shown that combination-BT is an effective method for achieving better dose distribution for advanced cervical lesions. Even manual optimization was able to bring about better coverage of the HR-CTV, with no significant increase of the dose to OARs in combination-BT. In addition, IPSA with combination-BT has resulted in significant sparing of normal tissues without compromising CTV coverage, compared with manual optimization.

Transperitoneal template techniques were commonly used as interstitial treatment methods for locally advanced tumor [18–21]. However, using these methods, the applicators have to be left for a few days after implantation. On the other hand, combination-BT is done for each brachytherapy session. The applicators do not need to be left and patients can be treated on an outpatient basis. In addition, there is no concern about interfractional differences in the needle positions, since the plastic applicators are inserted each time. Similar techniques have already been reported from Vienna University and Gunma University. Kirisits *et al*. [22] used their modified ring applicator. Their combined intracavitary and interstitial BT provides a prescription dose of up to 15 mm lateral of point A. Wakatsuki *et al*. [23] inserted a needle applicator from the vaginal vault inside the ovoid. Unlike the Vienna University technique, our technique does not need a particular applicator. In addition, our technique can place needles in a greater variety of positions and make various dose distributions adapted to the tumor shape, as in the method of Wakatsuki *et al*.

Comparison of IPSA with other optimizing methods, such as graphical optimization and dose-point optimization for interstitial template brachytherapy, has previously been reported [7, 14–16]. Most of the earlier investigations were carried out in prostate and cervical cancer, using a template technique [9–16]. Their reports described a significant sparing of normal tissues without compromising tumor coverage. Although there are no reports on an appropriate optimizing method for combination-BT, the same tendency is demonstrated in this study.

Although combination-BT and IPSA techniques result in superior plans in terms of HR-CTV coverage, and sparing normal tissues with ease, caution is recommended in the general application of IPSA. Because the number of applicators is limited, source dwell time in each applicator is longer than that with the template technique and more applicators. Therefore, combination-BT techniques will have a greater high-dose-volume than template techniques with more applicators. Some reports showed that complications increase with a greater V_PD_ [24, 25]. Although the V_PD_ and V_2PD_ were significantly less than using the conventional Manchester system, the location of the high-dose area differed from that in the conventional Manchester system. Some clinical reports have indicated tolerance and a good local control rate upon using IPSA. Thibault *et al*. [26] reported on their 43 patients' clinical experiences of ISBT using IPSA for a locally advanced population unsuitable for ICBT. The two-year local control (LC) rate for primary cancer was 87%, and Grade 3/4 late morbidity occurred in 12 patients. Their high incidence of severe late toxicities was primarily related to vaginal necrosis. They believe that those complications may have been related to the learning curve of inserting the needles. Kim *et al*. [27] reported on 51 patients' experiences. There were no toxicities of Grade 4 or greater, and the frequencies of Grade 3 acute and late toxicities were 4% and 2%, respectively. Local recurrence developed in only two patients. Although their initial clinical outcomes were tolerable, careful attention should be paid to minimize the dose to normal tissues. In addition, longer follow-up on the side effects is needed for the application of combination-BT and IPSA in clinical practice.

## CONCLUSION

In conclusion, to our knowledge, this is the first report on the employment of the IPSA technique in HDR combination-BT planning. Combination-BT achieved better tumor coverage and plans using IPSA and provided significant sparing of normal tissues without compromising CTV coverage compared with manual optimization.

## FUNDING

Part of this study was financially supported by the Cancer Research Development Fund (23-A-13) of the National Cancer Center and the Cancer Clinical Research Fund of the Ministry of Welfare, Health and Labor.
